# Lipopolysaccharide-Induced Transcriptional Changes in LBP-Deficient Rat and Its Possible Implications for Liver Dysregulation during Sepsis

**DOI:** 10.1155/2021/8356645

**Published:** 2021-12-30

**Authors:** Zhixiang He, Zichen Song, Leilei Meng, Wenhui Cheng, Fan Huang, Mao Zheng, Wenhui Xu, Rong Xiao, Haoshu Fang, Yaling Zhu

**Affiliations:** ^1^Department of Pathophysiology, Anhui Medical University, Hefei 230000, China; ^2^The First Clinical Medical College, Anhui Medical University, Hefei 230000, China; ^3^Laboratory Animal Research Center, College of Basic Medical Science, Anhui Medical University, Hefei 230000, China; ^4^Department of General Surgery, First Affiliated Hospital of Anhui Medical University, 218 Road, Hefei, China; ^5^Department of Endocrinology, First Affiliated Hospital of University of Science and Technology of China, Hefei 230000, China

## Abstract

Sepsis is an organ dysfunction caused by the dysregulated inflammatory response to infection. Lipopolysaccharide-binding protein (LBP) binds to lipopolysaccharide (LPS) and modulates the inflammatory response. A rare systematic study has been reported to detect the effect of LBP gene during LPS-induced sepsis. Herein, we explored the RNA sequencing technology to profile the transcriptomic changes in liver tissue between LBP-deficient rats and WT rats at multiple time points after LPS administration. We proceeded RNA sequencing of liver tissue to search differentially expressed genes (DEGs) and enriched biological processes and pathways between WT and LBP-deficient groups at 0 h, 6 h, and 24 h. In total, 168, 284, and 307 DEGs were identified at 0 h, 6 h, and 24 h, respectively, including *Lrp5*, *Cyp7a1*, *Nfkbiz*, *Sigmar1*, *Fabp7*, and *Hao1*, which are related to the inflammatory or lipid-related process. Functional enrichment analysis revealed that inflammatory response to LPS mediated by *Ifng*, *Cxcl10*, *Serpine1*, and *Lbp* was enhanced at 6 h, while lipid-related metabolism associated with *C5*, *Cyp4a1*, and *Eci1* was enriched at 24 h after LPS administration in the WT samples. The inflammatory process was not found when the LBP gene was knocked out; lipid-related metabolic process and peroxisome proliferator-activated receptor (PPAR) signaling pathway mediated by *Dhrs7b* and *Tysnd1* were significantly activated in LBP-deficient samples. Our study suggested that the invading LPS may interplay with LBP to activate the nuclear factor kappa B (NF-*κ*B) signaling pathway and trigger uncontrolled inflammatory response. However, when inhibiting the activity of NF-*κ*B, lipid-related metabolism would make bacteria removal via the effect on the PPAR signaling pathway in the absence of LBP gene. We also compared the serum lactate dehydrogenase (LDH) and alkaline phosphatase (ALP) levels using the biochemistry analyzer and analyzed the expression of high mobility group box 1 (HMGB1) and cleaved-caspase 3 with immunohistochemistry, which further validated our conclusion.

## 1. Introduction

Sepsis is a life-threatening disorder accompanied by organ dysfunction [1], which remains the leading cause of mortality in critically ill patients [2]. Despite years of intensive study and advances in pathogenesis and supportive care, sepsis is still an enigmatic disease and a horrendous financial burden for the healthcare system [3].

Lipopolysaccharide (LPS), a major constituent of the outer cell wall of gram-negative (GN) bacteria, is considered to be the most important activator in the pathogenesis of sepsis, of which minute amounts can initiate the molecular mechanisms of the innate immune response [4, 5]. According to the previous study, the dysregulated inflammatory response initiated by the interaction between lipopolysaccharide-binding protein (LBP) and LPS is closely related to the development of sepsis [6]. LBP is a class I acute-phase protein primarily synthesized by hepatocytes [7]. It firstly recognizes LPS released from infecting pathogens by forming a high-affinity complex. The LBP-LPS complex is transferred to the cluster of differentiation 14 (CD14) and toll-like receptor (TLR) 4 to trigger the release of inflammatory cytokines [8]. Additionally, increasing evidence indicated that lipid metabolism is correlated with the host's proinflammatory status. The inflammatory response is promoted by both obesity and high-fat meals, which may alter the intestinal barrier via affecting the gut microbiota to translocate LPS into the bloodstream [9, 10]. LBP acts catalytically to facilitate binding of LPS to lipoproteins such as very low-density lipoprotein, low-density lipoprotein, and high-density lipoprotein, which also represent an important mechanism in host defense to inactivate with LPS [11, 12].

Previous reports indicate that the liver plays a central role in the modulation of LPS response in sepsis, which is responsible for the bacterial clearance and also mediates the LPS-induced systemic inflammatory injury [13, 14]. To further detect the transcriptomic changes in liver tissue and investigate the role of LBP in LPS-induced inflammatory response, we explored the RNA sequencing technology to compare gene expression profiling between LBP-deficient groups and WT groups at 0 h, 6 h, and 24 h after LPS infection and identified candidate genes, biological processes, and signal pathways which are functionally related to sepsis, providing new clues for clinical treatment.

## 2. Materials and Methods

### 2.1. Experimental Animals and Tissue Collection

A total of 18 male SD and LBP^−/−^ rats (body weight 230 ± 20 g) were used in this study. SD rats were originally provided by Beijing Vital River Laboratory Animal Technology Co., Ltd., and LBP^−/−^ rats were purchased from the Nanjing Biomedical Research Institute of Nanjing University, which had the same genetic background as SD rats. All animals were housed under standard animal care conditions and had free access to water and rat chow ad libitum. Thick corn cob padding and nest material were used for enrichment of housing environment. Animals were acclimatized for 7 days before treatment. All procedures were carried out according to the Animal Welfare legislation of China. Animal experiments were approved by the ethics committee of Anhui Medical University. All the treatments were performed under inhalation anesthesia using vaporized isoflurane (Raymain, Shanghai, China). The anesthesia was induced in a chamber and maintained using a face mask with a 0.5 L/min oxygen flow mixed with 3% isoflurane. The injection and operation started when the rat had no more pain reflexes, e.g., no response to clamping the skin using surgical forceps.

SD rats and LBP^−/−^ rats were divided into the control and treated groups, respectively (*n* = 9 per group), and anesthetized. Rats were challenged with a sublethal LPS injection (2 mg/kg, intravenous injection, E. coli serotype O55:B05 type, Sigma-Aldrich, St. Louis, USA). Meloxicam (0.2 mg/kg, subcutaneous injection, TargetMol) was administered to achieve the postoperative analgesia. Penicillin was not applied considering no open wound and low possibility of infection within 24 h after LPS administration. At 0 h (*n* = 3), 6 h (*n* = 3), and 24 h (*n* = 3) after LPS administration, rats were sacrificed under 5% isoflurane (Raymain, Shanghai, China). Blood was taken from the inferior vena cava, and the liver tissues were collected and used for succeeding transcriptome sequencing and data analysis. Subsequently, all the rats were euthanatized with 5% isoflurane (Raymain, Shanghai, China).

Phenotypic values were presented as the mean ± standard deviation (*M* ± SD). Statistical comparisons of phenotypic values between the experimental and normal groups were conducted by the Student *t*-test. The statistical difference was considered significant at *P* < 0.05 and highly significant at *P* < 0.01.

### 2.2. RNA Extraction and Sequencing

Total RNA was extracted from 100 mg of liver tissue from three LBP-deficient experimental individuals and three normal individuals using the RiboPure kit (Ambion, Austin, USA) according to the manufacturer's protocol. The RNA integrity was assessed by an Agilent Bioanalyzer 2100 and RNA Nano 6000 Lab chip kit (Agilent Technologies, USA). Sequencing libraries were generated using the NEBNext Ultra™ Directional RNA Library Prep Kit (Illumina, USA) following the manufacturer's recommendations, and index codes were added to attribute sequences to each sample. Then, the paired-end sequencing of the libraries was constructed on a Hi-Seq 4000 platform (Illumina, USA) via Novogene (Novogene, USA). The resultant data will be deposited at the NCBI Sequence Read Archive (SRA) database upon acceptance.

### 2.3. Mapping, Assembling, and Annotation of Sequence Reads

First, the RNA-seq reads were discriminated based on the indexing adaptors. Low-quality reads and those containing ploy-N were then removed from raw data using FastQC v0.11.7 (http://www.bioinformatics.bbsrc.ac.uk/projects/fastqc). Next, the filtered reads were mapped against the chicken reference genome Gallus_gallus-5.0 (Ensembl) using STAR-2.5.3a [15], a fast splice junction mapper for short and long RNA-seq reads to a reference genome using uncompressed suffix arrays. Parameters of STAR were set to only allow unique alignment to the reference genome. Transcripts were assembled and quantified by Stringtie-1.3.3b [16]. In addition, we explored S-MART (http://urgi.versailles.inra.fr/Tools/S-MART) to calculate the distribution of reads mapped to exons, introns, and 1 kb upstream/downstream of the annotated genes. To count the number of reads uniquely mapped to an exon, featureCounts was used with “gene” as a feature and not strand-specific [17]. Since low expressed genes are more vulnerable to measurement errors, we removed low expressed genes whose counts were lower than 2 in 90% of samples. And then, FPKM (expected number of Fragments Per Kilobase of transcript sequence per Million base pairs sequenced) of each gene was calculated based on the length of the gene and read count mapped to this gene. FPKM considers the effect of sequencing depth and gene length for the read counts at the same time and is currently the most commonly used method for estimating gene expression levels from RNA-seq data [18].

### 2.4. Hierarchical Clustering

After quality control, we investigated sample heterogeneity between wild and LBP-deficient liver transcriptome data by performing unsupervised hierarchical cluster analysis. Raw *z*-scores were firstly calculated from counts of wild and LBP-deficient samples and then subjected to agglomerative hierarchical clustering analysis based on Ward's method and Euclidean distance. Bioinformatics analysis was performed in R version 3.5.1, and a heat map was generated by the pheatmap package from CRAN R-project (https://CRAN.R-project.org/package=pheatmap).

### 2.5. Differential Gene Expression Analyses

Differential expression analyses of the LBP^−/−^ experimental and normal groups were performed using the DESeq2 R package [18]. It provides statistical routines for determining DEGs from digital gene expression data using a model based on the negative binomial distribution. The resulting *P* values were adjusted using Benjamini and Hochberg's approach for controlling the false discovery rate [19]. Genes with adjusted *P* value less than 0.05 and log2(fold change) greater than 1.5 were assigned as DEGs.

### 2.6. Gene Ontology and Pathway Enrichment Analyses

DAVID (https://david-d.ncifcrf.gov/) and PANTHER (http://www.pantherdb.org/) were executed to identify overrepresented Gene Ontology (GO) terms and pathways of the DEGs. GO terms with a corrected *P* value less than 0.05 were considered significantly enriched by DEGs. KEGG is a database resource for understanding high-level functions and utilities of the biological system, such as the cell, the organism, and the ecosystem, from molecular-level information, especially large-scale molecular datasets generated by genome sequencing and other high-throughput experimental technologies (http://www.genome.jp/kegg/). We used the KOBAS software (http://kobas.cbi.pku.edu.cn) to test the statistical enrichment of DEGs in KEGG pathways.

### 2.7. Quantitative Polymerase Chain Reaction (qPCR)

Total RNA was isolated from liver tissues using the TRIzol Reagent (CoWin Biosciences, China) following the manufacturer's instructions. Complementary DNA synthesis was conducted using the First Strand cDNA synthesis kit K1622 (Thermo Fisher, USA). qPCR was carried out by using C1000 touch thermal cycler CF-X96™ (Bio-rad, USA) with SYBR Select master mix (Catalog number: 4472908) (Thermo Fisher, USA) and rat gene-specific primers (Table S1). The amplification consists of 95°C for 3 min, followed by 40 cycles of 95°C for 10 s and 60°C for 30 s. The relative mRNA expression levels were calculated according to the 2^-*ΔΔ*Ct^ method and normalized using glyceraldehyde-3-phosphate dehydrogenase (GAPDH) (Table S1).

### 2.8. Liver Enzymes

To investigate the hepatocellular injury in normal and LBP-deficient rats after LPS induction, we took venous blood from the cavity and measured the levels of serum lactate dehydrogenase (LDH) and alkaline phosphatase (ALP) using an Automated Chemical Analyzer (Bayer Advia 1650; Leverkusen, Germany).

### 2.9. Immunohistochemistry

Fresh liver tissues were fixed in 10% formalin for 24 h and embedded in paraffin. Each liver tissue sample was sectioned at 4 *μ*m thicknesses using a Leica microtome (Leica Microsystems, Buffalo Grove, IL, USA). The analysis of the high mobility group box 1 (HMGB1) and cleaved-caspase 3 proteins was performed using polyclonal rabbit anti-HMGB1 antibody (Abcam, Cambridge, UK, 1 : 500) and polyclonal antibody of cleaved-caspase 3 (Cell Signaling, Beverly, MA, USA, 1 : 100), respectively. The staining was documented at a magnification of 400x. The percentage of hepatocytes with only nucleus HMGB1 staining out of the total number of hepatocytes was calculated.

## 3. Results

### 3.1. Mapping and Annotation of RNA Sequencing Reads

**Figure 1:**
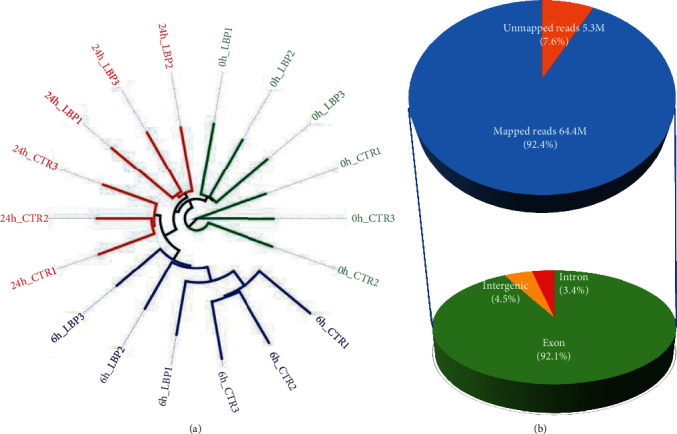
Summary of RNA-seq data of normal and LBP-deficient rats. (a) Neighbor-joining tree of normal and LBP-deficient samples treated with LPS for the times indicated (0 h, 6 h, and 24 h). Each condition has 3 replicates. Logarithm transformed counts from the RNA-seq dataset were computed for sample correlation by Pearson's correlation. CTR: normal rat; LBP: LBP-deficient rat. (b) A pie chart of average mapping statistics involving RNA-seq data.

The RNA sequencing technique integrated with bioinformatics analysis was used to characterize alteration in liver gene expression between WT and LBP-deficient samples triggered by LPS-induced systemic inflammation. We obtained about 69.7 million (M) of 150 bp paired-end reads for each sample (ranging from 57.2 to 107.2 million reads) (Table S2). After ambiguous mapping (allowing for multihits) via STAR-2.5.3a [15], a total of ~64.4 M reads for each sample were mapped against the rat reference genome Rattus_norvegicus.Rnor_6.0 (Ensembl, ftp://ftp.ensembl.org/pub/release-96/fasta/rattus_norvegicus) (Figure 1(b), Table S2). Among the mapped reads, 92.1% of these reads were mapped to exonic regions, 4.5% mapped intergenic regions, and 3.4% mapped intronic regions (Figure 1(b)).

To evaluate the segregation between WT and LBP-deficient samples during the different times after LPS administration, we conducted the neighbor-joining tree of samples based on the expression of all genes. As shown in Figure 1(a), clear divergence between the time of LPS treatment (0 h, 6 h, and 24 h) was observed in this tree, and WT and LBP-deficient rats also defined their respective separate clades, suggesting high fidelity of our RNA-seq data.

### 3.2. Systemic Administration of Bacterial LPS Induces Global Changes in the Liver Transcriptome

**Figure 2:**
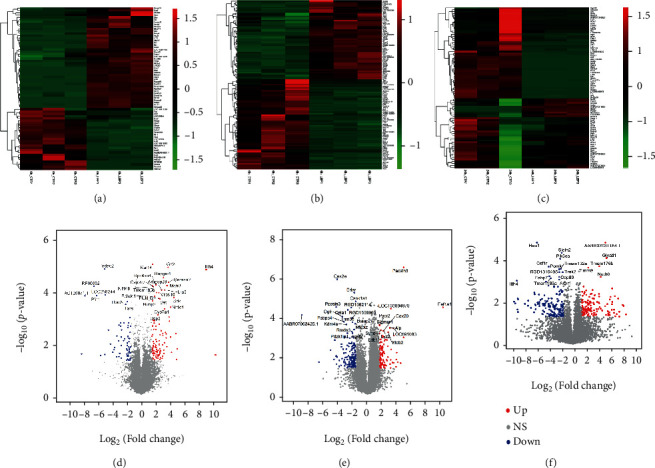
Distinct transcriptional signature between WT and LBP-deficient rats. (a–c) Transcription profiles of significant differentially expressed genes (DEGs) with log2(fold change) larger than 1.5 at *P* value < 0.01 at 0 h, 6 h, and 24 h, respectively. The labeling condition and DEGs were adapted as previous panels. (d–f) The volcano plot of LPS-induced transcriptional changes between normal and LBP-deficient rats with the time of 0 h, 6 h, and 24 h, respectively. Differential expression genes with log2(fold change) larger than 1.5 at *P* value < 0.05 were colored with blue (downregulated) and red (upregulated).

To further characterize the DEGs from our RNA-seq data, an analysis was performed to screen DEGs with a *P* value less than 0.05 and log2(fold change) higher than 1.5 using the DESeq2 R package [18] (Figure 2). In total, we identified 168, 284, and 307 significantly alternative genes, respectively, during the time of 0 h, 6 h, and 24 h between the normal and LBP-deficient samples. Then, we clustered these differentially expressed genes (DEGs) via a hierarchical heat map (Figures 2(a)–2(c)) to depict the differential expression gene profile between the normal and LBP^−/−^ rats. The most significantly DEGs with *P* value < 0.001 and log2(fold change) > 1.5 were labeled in the volcano plots (Figures 2(d)–2(f), Table S3). Among that, *Lrp5* [20], *Cyp7a1* [21], *Nfkbiz* [22], *Sigmar1* [23], *Fabp7* [24], and *Hao1* [25] (Figure S1) have been reported in the inflammatory response and lipid metabolic process, suggesting that these genes may play an important role in modulating sepsis-induced system inflammation in WT and LBP-deficient rats after LPS injection.

### 3.3. Gene Annotation and Gene Ontology Analyses of DEGs

**Figure 3:**
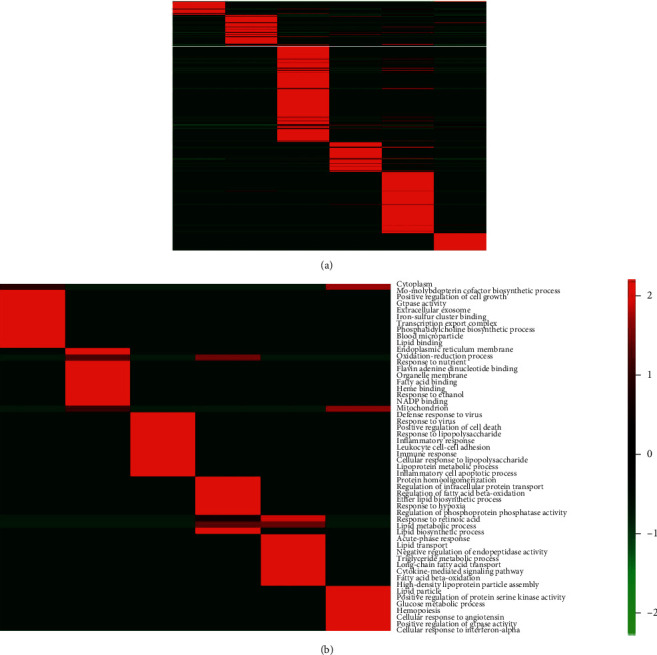
Association of differential genes with functional Gene Ontology (GO) terms. (a) Full enrichment dataset heat map for GO terms from WT and LBP^−/−^ at 0 h, 6 h, and 24 h after LPS challenge. (b) Ten representative differentially enriched GO terms.

To further study significantly overrepresented Gene Ontology terms involving these DEGs during 0 h, 6 h, and 24 h after LPS administration, functional annotations were performed with the DAVID Bioinformatics Resources 6.7 (https://david-d.ncifcrf.gov/), respectively [26]. Selecting from the full enrichment datasets (Figure 3(a)), we found ten representative terms with the exhibition of strong differential enrichment patterns mainly related to inflammatory response, immune response, and lipid metabolic processes (Figure 3(b)). Further, to detect the most associated DEGs during those biological processes between healthy and LBP-deficient groups, we exhibited associated genes evolved in the representative terms and pathways (Table 1). Interestingly, we found that in the normal rats, LPS strongly upregulated genes involved in the processes of the inflammatory response and immunomodulation including *Ifn-γ* [27], *Cxcl10* [28], *Serpine1* [29], and *Lbp* [30] (Figure S2a-d) at 6 h after LPS injection, then proceed in lipid metabolic response including *C5* [31], *Cyp4a1* [32], and *Eci1* [33] (Figure S2e-g) at 24 h (Table 1(a)). And the enriched pathways were in accordance with the results of Gene Ontology (Table 1(b)), which revealed that inflammatory pathways containing the toll-like receptor signaling pathways and natural killer cell-mediated cytotoxicity were enhanced in the normal groups at 6 h and lipid-related metabolism of peroxisome proliferator-activated receptor (PPAR) signaling pathway was enriched at 24 h after LPS administration. Conversely, the functional enrichment of DEGs in LBP-deficient groups predominantly activated the lipid metabolic response instead of inflammatory or immunological response during the first two time points, enriching some upregulated genes such as *Dhrs7b* [34] and *Tysnd1* [35] (Figure S2h-i). And the PPAR signaling pathway was significantly overrepresented at the first two time points, which suggests modulating inflammation and bacterial killing after LPS challenge with the deficiency of the LBP gene [36]. Interestingly, the DEGs both in the healthy and LBP^−/−^ groups were overrepresented in the processes of lipid metabolic and repeatedly enriched genes of *Eci1*, *Pnpla3* [37], *Apoa5* [38], and *Fabp1* [39] (Figure S2j-l).

### 3.4. A Proposed Model of the Roles of NF-*κ*B and PPAR Signaling Pathways in the WT and LBP-Deficient Rats after LPS Challenge

**Figure 4:**
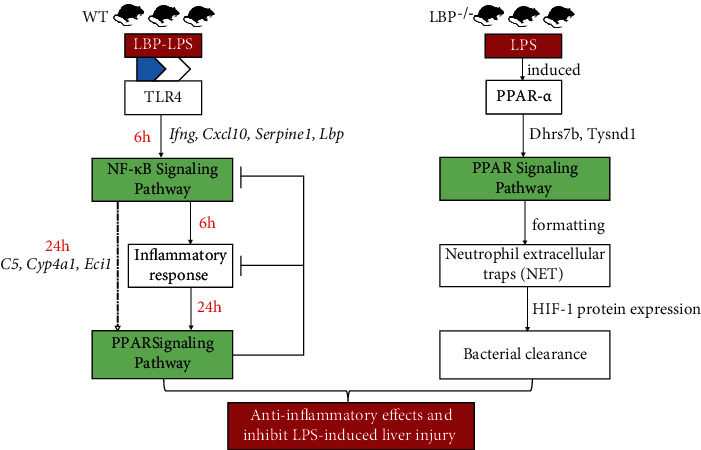
A proposed model of the WT and LBP-deficient rats after LPS administration. The upregulation of *Ifng*, *Cxcl10*, *Serpine1*, and *Lbp* in WT rats triggers NF-*κ*B signaling pathway-induced inflammation response at 6 h after LPS injection, while the PPAR signaling pathway plays a part in bacterial clearance via the formation of NET by highlighted genes of *C5*, *Cyp4a1*, and *Eci1* at 24 h after LPS administration in SD rats and of *Dhrs7b* and *Tysnd1* in the LBP^−/−^ rats.

Based on the biological functions of the abovementioned genes and previous studies of nuclear factor kappa B (NF-*κ*B) and PPAR signaling pathways, we presented a proposed model for the development of sepsis in rats (Figure 4). At 6 h, the upregulation of *Ifng*, *Cxcl10*, *Serpine1*, and *Lbp* in WT rats triggers NF-*κ*B signaling pathway-induced inflammation response after LPS injection. And the activation of the NF-*κ*B signaling pathway is responsible for modulating the immune reaction via enhanced biosynthesis of large quantities of proinflammatory molecules, including cytokines and adhesion molecules, which frequently induce sepsis and cause tissue damage when their production is dysregulated and excessive [40, 41].

At 24 h, the PPAR signaling pathway was found and may function as bacterial clearance via the formation of NET by highlighted genes of *C5*, *Cyp4a1*, and *Eci1* in SD rats and enhanced *Dhrs7b* and *Tysnd1* in the LBP^−/−^ rats after LPS administration. Just as reports revealed, PPARs are a large superfamily of nuclear receptors and incorporate three isoforms (PPAR-*α*, PPAR-*β*, and PPAR-*γ*), which are broadly involved in the regulation of metabolism, especially associated with lipid and glucose homeostasis [42, 43]. In the process of activating pathway, PPAR-*α* negatively regulates the activity of the transcription factor to inhibit the expression of proinflammatory mediators such as tumor necrosis factor alpha (TNF-*α*), interleukin 12 (IL-12), and adhesion molecules which result in anti-inflammatory outcomes in the setting of sepsis induced by LPS [44].

Together, the proposed model reflects that invading LPS may interplay with LBP to activate the NF-*κ*B signaling pathway and trigger uncontrolled inflammatory response. However, when inhibiting the activity of NF-*κ*B, lipid-related metabolism would make bacteria removal via the effect on the PPAR signaling pathway in the absence of LBP gene.

### 3.5. qPCR Validation of Related Differentially Expressed Genes

**Figure 5:**
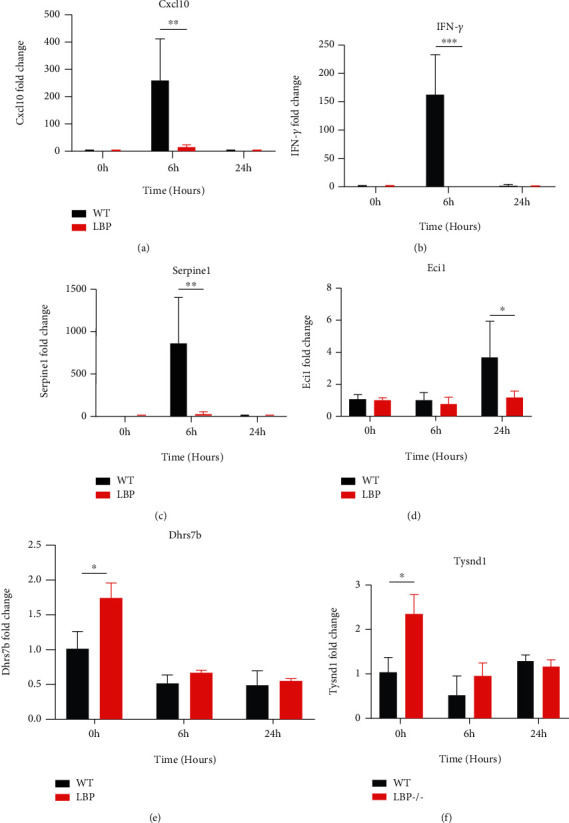
qPCR verification of DEGs in liver tissues between the normal and LBP-deficient rats in LPS-induced sepsis. Expression levels of interested genes from the transcriptome dataset were verified by qPCR analysis. The relative gene expression was determined using the 2^-*ΔΔ*Ct^ method with the normalization of GAPDH. (a–c) The inhibition of inflammation-related genes including *Cxcl10*, *Ifn-γ*, and *Serpine1*. (d–f) The upregulation of *Eci1*, *Dhrs7b*, and *Tysnd1* involved in the process of lipid metabolism. ^∗^*P* < 0.05, ^∗∗^*P* < 0.01, and ^∗∗∗^*P* < 0.001 vs. normal rats.

To validate our results from RNA-seq analysis, we further adopted quantitative PCR to examine the DEGs related to inflammatory response and lipid metabolic process, including *Ifn-γ*, *Cxcl10*, *Serpine1*, *Eci1*, *Dhrs7b*, and *Tysnd1*. As demonstrated in Figures 5(a)–5(c), the expression levels of inflammation-related genes of *Cxcl10*, *Ifn-γ*, and *Serpine1* were significantly reduced in the LBP-deficient rats at 6 h after LPS injection compared to the normal rats (*P* < 0.05), while the lipid-related gene of *Eci1* in the LBP-deficient rats at 24 h after LPS administration was significantly elevated (Figure 5(d)). *Dhrs7b* and *Tysnd1* also upregulated in the LBP^−/−^ rats at 0 h after LPS challenge (Figures 5(e) and 5(f)). Collectively, our independent qPCR approach is generally concordant with previous RNA-seq results.

### 3.6. Hepatocellular Damage in Normal and LBP-Deficient Rats after LPS Administration

**Figure 6:**
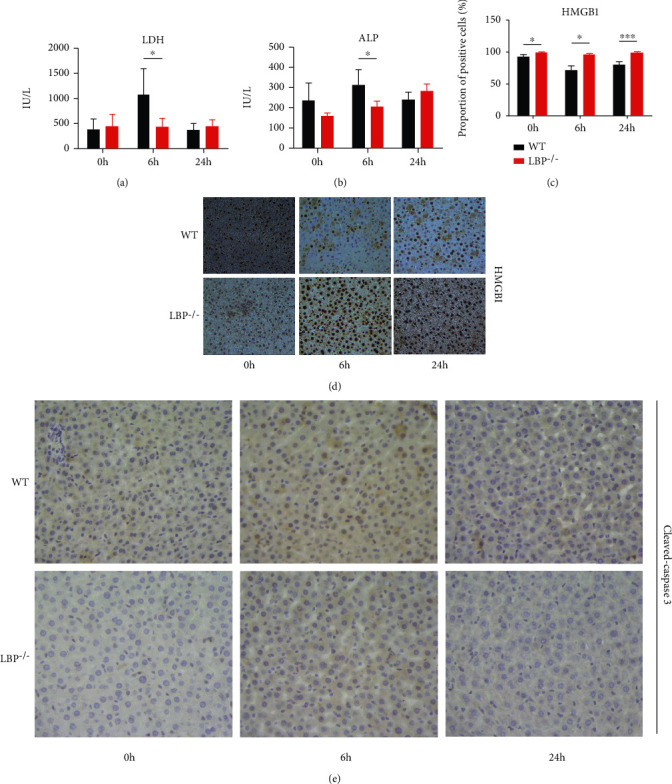
Expression levels of inflammatory indicators in wild-type and LBP-deficient groups after LPS injection. (a, b) Levels of liver enzymes of LDH (a) and ALP (b) levels in serum samples collected at 0 h, 6 h, and 24 h after LPS challenge. Serum LDH and ALP levels were analyzed as a measure of hepatocellular injury. Data are shown as means and standard deviations (*n* = 3 per group at each time point). (c) The percentage of hepatocytes with only nucleus HMGB1 staining out of the total number of hepatocytes was calculated. Data were shown as the mean ± SD. (d) HMGB1 cellular location of liver tissues in the normal rats and LBP^−/−^ rats at 0 h, 6 h, and 24 h after LPS administration. Original magnification ×400. (e) The expression levels of cleaved-caspase 3 in the wild and LBP^−/−^ rats at multiple time points after liver LPS injection were assessed using IHC. Original magnification ×400. ^∗^*P* < 0.05, ^∗∗^*P* < 0.01, and ^∗∗∗^*P* < 0.001 vs. wild type, significantly different from the wild-type groups.

Inferior vena cava blood was collected from 3 normal and 3 LBP-deficient rats after LPS induced at 0 h, 6 h, and 24 h, respectively; then, we analyzed the serum levels of LDH and ALP. In the normal groups, the serum LDH and ALP levels reached a peak at 6 h after LPS administration (Figure 6(a)), suggesting the most severe liver injury. In contrast, less hepatocellular damage was observed in consistence with the obvious decreases in serum levels of LDH and ALP in LBP-deficient samples (Figures 6(a) and 6(b)).

### 3.7. Immunohistochemical Examination for the Expressions of Inflammatory Indicators in Liver Tissues

We proceeded with immunohistochemical staining of typical inflammatory mediators to evaluate the inflammatory injury in the normal and LBP^−/−^ rats at multiple time points after LPS injection. As shown in Figure 6(d), the normal rats showed more HMGB1 translocation from nucleus to cytoplasm after LPS challenge. The percentage of nucleus positive HGBM1 hepatocytes was 74.68 ± 23.33% at 6 h (vs. LBP-deficient rats: 96.28 ± 4.52%, *P* < 0.05) and then increased at 24 h (79.97 ± 20.94%, vs. LBP-deficient rats: 99.51 ± 1.13%, *P* < 0.001) (Figure 6(c)). Then, we characterized the occurrence of apoptosis after LPS injection by cleaved-caspase 3 staining. Cleaved-caspase 3 expression was barely detectable in LBP^−/−^ rats, whereas hepatocytes of the normal rats were strongly positive (Figure 6(d)). In conclusion, the above results elucidated the attenuated inflammatory injury of hepatocytes after LPS challenge when knocking out the LBP gene.

## 4. Discussion

### 4.1. Potent Alterations of Pathways in LBP-Deficient Rats in comparison with the Normal Rats after LPS Administration

Although LPS-induced sepsis has a system-wide impact, considering that the LBP is synthesized by the liver tissue, plays a decisive role in mediating the LPS-induced inflammatory response, and determines the severity of systemic injury, we investigated the role of LBP at the background of liver tissue in this study [45]. We adopted RNA sequencing to confirm that the LBP expression level was elevated after LPS treatment *in vivo* (Figure S2d), following the previous research that LBP delivers LPS to CD14 and TLR4 and finally triggers a cascade of events including the translocation of NF-*κ*B to the nucleus and the initiation of the production and release of inflammatory cytokines via the activation of the TLR-4 signaling pathway [46].

PPAR-*α* stimulated with correlative ligands performed anti-inflammation activity via downregulating NF-*κ*B actions and subsequently inhibiting the expression of inflammatory mediators, such as TNF-*α*, IL-12, and adhesion molecules [36]. It could be deemed that the diminished liver inflammation and injury in the normal groups at 24 h may be performed by modulating the inflammatory response through the PPAR-*α* signaling pathway. Additionally, combined with the enrichment results of functional annotations and pathways, we surmised that given at the time of resuscitation, LBP-deficient rats would reduce liver injury by enhancing bacterial clearance through the PPAR signaling pathway, as reported that the activation of PPAR-*α* increased the formation of neutrophil extracellular traps (NET) containing neutrophil, histones, and granule proteins, which may potentially propose a protective mechanism of bacterial elimination in the LBP-deficient group [36].

HMGB1, acting as an inflammatory mediator, is responsible for the production and release of proinflammatory cytokines in many inflammatory and infectious disorders, including acute lung injury, liver ischemia-reperfusion injury, and sepsis [47–51]. Stimulated by LPS, HMGB1 can translocate from nucleus to cytoplasm [51, 52]. In this study, HMGB1 translocation occurred most in the normal rats at 6 h and then decreased at 24 h, consistent with the results of RNA-seq and the levels of liver enzymes (Figures 6(c) and 6(d)). And the cleaved-caspase 3 level was significantly decreased in LBP^−/−^ rats, indicating less hepatocyte damage compared to the normal rats [53, 54]. In short, liver inflammatory injury was relieved in LBP-deficient rats in comparison to the normal rats after LPS administration.

### 4.2. Poor Effects of Anti-Inflammatory Therapies during Sepsis

Our findings support that inflammatory response is closely associated with liver injury, which can further demonstrate that dysregulated inflammatory response exerts a crucial part in the development of sepsis [6]. However, during the last decades, the effects of many clinical trials testing anti-inflammatory approaches on patients with sepsis were rather disappointing. Gordon et al. [55] suggested that AZD9773, a polyclonal fragment antibody which has the effect of decreasing the concentration of TNF-*α* in circulation, was short of clinical benefit. Steven et al. [56] demonstrated that Eritoran did not improve survival among patients with sepsis shock as the antagonist of the MD2-TLR4 receptor for treatment. The administration of a high dose of corticosteroids, anti-inflammatory agents that globally depress the activity of the immune system and reduce the damage from cytokines and neutrophils, also failed to bring about improving outcomes for patients with sepsis [57]. Thus, it can be possible to conclude that the development of sepsis in humans is not merely the modulation of the inflammatory response; more comprehensive exploration concerning complicated molecular mechanisms requires undertaking for more effective clinical treatment.

### 4.3. The SNPs in LBP and the Potential of LBP as a Biomarker in Clinical Application

The mechanisms mediated by LBP are a crucial player in the production of sepsis and related metabolic disorders, which makes it rational to suppose that single nucleotide polymorphisms (SNPs) within LBP gene might be determinants for interindividual susceptibility. Eckert et al. [58] previously found that the rs2232613 polymorphism, leading to the substitution of proline with leucine at position 333 of LBP protein, was associated with a reduced ability to bind LPS or induce cytokines *in vitro*. The phenotype of individuals carrying the rs2232618 (Phe436Leu) had significant relevance with the higher incidence of sepsis and multiple organ dysfunction [59]. Another study also showed that susceptibility to severe sepsis was strongly correlative with a common haplotype from the 5′-flanking region of the LBP gene [60]. Additionally, a foregoing study reported that the rs2232592 polymorphism, located in the intron of LBP, was significantly related to type 2 diabetes [61]. Briefly, polymorphisms within the LBP gene might have an intensive association with sepsis and metabolic risk, which emphasize the immense potential of LBP in clinical application.

It has been performed that the upregulation of LBP was widely observed in patients with severe infectious diseases [62], and increased circulating LBP levels are correlative with the severity of sepsis [63], suggesting that LBP may serve as a valuable biological marker for diagnosis and prognosis of patients with sepsis. However, previous reports showed that LBP provided little clinical favorable information. Compared to other traditional biomarkers, such as procalcitonin and C-reactive protein, LBP has a moderate degree of diagnostic accuracy for sepsis [64]. Similarly, Sakr et al. [62] demonstrated that LBP moderately discriminated patients without infection from patients with severe sepsis.

What is worth noting is that although LBP concentrations may weakly correlate with the severity and outcome of sepsis, circulating LBP was elevated when it came to obesity, metabolic syndrome (MetS), and type 2 diabetes in apparently healthy Chinese [65]. According to the investigation concerning the association between LBP levels and 6-year incident MetS, Liu et al. [66] suggested that LBP was positively correlative with the increased 6-year risks of MetS among middle-aged and older Chinese. Besides, higher LPS or LBP concentrations could be observed in diabetic subjects than in healthy controls [67]. In short, LBP might be a promising biomarker of metabolic endotoxemia, but future prospective studies are still recommended for elucidating the potential biological mechanisms.

## 5. Conclusions

Taken together, to the best of our knowledge, we present here the first comprehensive profile of gene expression between the healthy and LBP-deficient rats after LPS induced at multiple time points using RNA sequencing technology. With all these data surrounding the influence of sepsis evoked by acute administration of LPS, we reported a list of genes that tremendously altered the liver tissue. Most importantly, we emphasized the modulation of uncontrolled inflammatory response triggered by the NF-*κ*B signaling pathway and bacterial elimination via lipid-related metabolism with the effect of the PPAR signaling pathway, which has the potential reason for the alleviated inflammatory response and the attenuated liver damage and mortality of rats. And we also exhibited the proposed model to explain the genetic mechanisms in LBP^−/−^ rats after LPS challenge, which may have more biological and clinical implications. However, further and ongoing in vivo studies are still required to confirm the proposed model and the candidate genes to ultimately validate the functional role of these findings.

## Figures and Tables

**(a):** 

Category		GO	Terms	Associated genes	*P*-value
WT	0 h	GO:0006656	Phosphatidylcholine biosynthetic process	*APOA2, FABP5*	3.5E-02
		GO:0008289	Lipid binding	*APOA2, PACSIN3, FABP5*	4.2E-02
	6 h	GO:0051607	Defense response to virus	*IFIT3, IFIT2, IFNAR2, OASL, ZC3HAV1, IFNG, CXCL9, CXADR, CXCL10*	5.9E-06
		GO:0010942	Positive regulation of cell death	*CDKN1A, GZMBL2, ZC3H12A, GZMB, FAS, BCL2L11*	5.5E-05
		GO:0032496	Response to lipopolysaccharide	*SELP, TNFRSF9, JUN, SOCS1, SERPINE1, IL1RN, CXCL9, LBP, FAS, PCK1, CXCL10*	5.5E-05
		GO:0006954	Inflammatory response	*SELP, NFKBIZ, TNFRSF9, OLR1, C4B, CXCL9, ZC3H12A, FAS, CELA1, CXCL10*	4.4E-04
		GO:0007159	Leukocyte cell-cell adhesion	*SELP, OLR1, TNIP1, NT5E*	8.7E-04
		GO:0006955	Immune response	*RT1-A2, TNFRSF9, GZMBL2, CXCL9, GZMB, FAS, SECTM1B, RT1-BB, CXCL10*	9.9E-04
		GO:0071222	Cellular response to lipopolysaccharide	*SERPINE1, IFNG, ZC3H12A, LBP, ABCA1, CXCL10, ADAM9*	1.7E-03
		GO:0042157	Lipoprotein metabolic process	*OLR1, APOL9A, ABCA1*	1.8E-02
		GO:0006925	Inflammatory cell apoptotic process	*IFNG, FAS*	2.3E-02
	24 h	GO:0006953	Acute-phase response	*HNRNPK, IL1RN, ITIH4, LBP, LOC100911545*	1.9E-04
		GO:0006641	Triglyceride metabolic process	*APOE, APOA5, CYP2E1, SLC22A5*	3.4E-03
		GO:0006635	Fatty acid beta-oxidation	*ECI1, EHHADH, DECR1, CROT*	5.1E-03
		GO:0006629	Lipid metabolic process	*SLC16A1, HNF4A, APOE, IL1RN*	3.0E-02
		GO:0034380	High-density lipoprotein particle assembly	*APOE, APOA5*	5.9E-02
		GO:0006869	Lipid transport	*APOE, APOA5, LBP*	7.6E-02

LBP^−/−^	0 h	GO:0055114	Oxidation-reduction process	*FMO5, CYP4A2, D2HGDH, HSD17B2, GPX4, CYP7A1, CYP2C7, DPYD, CYP8B1, POR*	2.8E-03
		GO:0005504	Fatty acid binding	*ACOX2, CYP4A2, ADH4*	7.8E-03
		GO:0005739	Mitochondrion	*ACOX2, D2HGDH, TRMT1L, SDS, GPX4, ADH4, MRPS10, VDAC2, PTEN, GPT2, ACSF2, POR, LRP5*	4.9E-02
	6 h	GO:0055114	Oxidation-reduction process	*CYP4A2, PYCR2, PIR, CYP4F6, FASN, ADH6, NQO1, DHRS7B, DDO, CYP2A3, FDFT1*	1.2E-03
		GO:0031998	Regulation of fatty acid beta-oxidation	*TYSND1, CPT1A*	2.4E-02
		GO:0008611	Ether lipid biosynthetic process	*FASN, DHRS7B*	2.9E-02
		GO:0001666	Response to hypoxia	*CD38, RAMP2, HSP90B1, CLDN3, ANGPTL4*	4.2E-02
		GO:0043666	Regulation of phosphoprotein phosphatase activity	*HSP90B1, RCAN1*	4.7E-02
		GO:0006629	Lipid metabolic process	*APOE, ACLY, MID1IP1*	6.9E-02
	24 h	GO:0006006	Glucose metabolic process	*LOC108351137, ONECUT1, MYC, FABP5*	7.5E-03
		GO:1904385	Cellular response to angiotensin	*CYBA, MYC*	5.9E-02
		GO:0035457	Cellular response to interferon-alpha	*MNDA, MYC*	7.6E-02
		GO:0030097	Hemopoiesis	*SGPL1, CIAPIN1, CSF1R*	8.3E-02
		GO:0005739	Mitochondrion	*NADK2, ABCF2, NUDT6, MRPS12, MRPS10, PLGRKT, QARS, LOC100359687, COMT, GLYATL1, FIS1,* *CYBA, SCCPDH, CHCHD10, ACSL1, KRAS, PARL, P2RY2, TIMM9, HEBP1, PXMP2, APEX1, MYC*	4.5E-04
		GO:0005811	Lipid particle	*SCCPDH, PNPLA3, ANXA2*	6.5E-02

**(b):** 

Category		ID	Pathways	Associated genes	*P*-value
WT	0 h	/	/	/	/
	6 h	rno05320	Autoimmune thyroid disease	*RT1-A2, RT1-CE7, GZMBL2, GZMB, FAS, RT1-BB*	8.0E-04
		rno04066	HIF-1 signaling pathway	*EGFR, CDKN1A, PFKFB3, HMOX1, SERPINE1, IFNG*	2.8E-03
		rno04060	Cytokine-cytokine receptor interaction	*IFNAR2, TNFRSF9, IFNG, CXCL9, EDAR, FAS, CXCL10*	1.2E-02
		rno04620	Toll-like receptor signaling pathway	*IFNAR2, JUN, CXCL9, LBP, CXCL10*	1.4E-02
		rno04650	Natural killer cell mediated cytotoxicity	*IFNAR2, GZMBL2, IFNG, GZMB, FAS*	1.4E-02
		rno04152	AMPK signaling pathway	*IRS2, CCND1, PFKFB3, CAB39, PCK1*	3.4E-02
	24 h	rno03320	PPAR signaling pathway	*CYP4A1, EHHADH, APOA5, FABP1, FABP7*	5.0E-03
		rno00071	Fatty acid degradation	*ECI1, CYP4A1, EHHADH, ADH6*	8.6E-03
		rno04060	Cytokine-cytokine receptor interaction	*CCL12, TNFSF10, LIFR, IL2RG, IL1A, CSF1R, ACVR1*	8.9E-03
		rno04931	Insulin resistance	*SREBF1, PTPRF, GFPT1, PIK3CA*	7.7E-02

LBP^−/−^	0 h	rno03320	PPAR signaling pathway	*ACOX2, CYP4A2, RXRA, CYP7A1, CYP8B1*	7.6E-04
		rno00140	Steroid hormone biosynthesis	*HSD17B2, CYP7A1, CYP2C7*	7.1E-02
	6 h	rno01100	Metabolic pathways	*CYP4A2, NAGS, NAT1, B3GALT4, ADH6, ACLY, FDFT1, CD38, PYCR2,* *UGT1A3, CYP4F6, GMPPA, FASN, LOC100912599, PAPSS1, CYP2A3*	1.6E-02
		rno00071	Fatty acid degradation	*CYP4A2, ADH6, CPT1A*	3.8E-02
		rno03320	PPAR signaling pathway	*CYP4A2, CPT1A, ANGPTL4*	9.1E-02
	24 h	rno01100	Metabolic pathways	*NADK2, SGPL1, QARS, COMT, PSPH, PNPLA3, GMPS, UMPS, ACSL1,* *GBE1, DPM3, LOC100912599, UGT2A3, ALG11, AMD1, FLAD1, CYP2C22*	3.2E-02

## Data Availability

The datasets generated and analyzed during the current study have been deposited at the GSA repository (https://bigd.big.ac.cn/gsa/browse/CRA002638).

## Supplementary Materials

Supplementary Materials Supplementary Table S1: primers for qPCR in the study. Supplementary Table S2: number of uniquely mapped reads for each sample. Supplementary Table S3: the labeled DEGs of volcano plot between normal and LBP-deficient rats with the time of 0 h, 6 h, and 24 h, respectively. Supplementary Figure S1: boxplots of interested DEGs related to inflammatory response and lipid metabolic process between normal and LBP-deficient rats after LPS injection. Supplementary Figure S2: boxplots of interested DEGs most associated with enriched GO terms and pathways.
