# Supplementary material for: Lipopolysaccharide-Induced Transcriptional Changes in LBP-Deficient Rat and Its Possible Implications for Liver Dysregulation during Sepsis
Source: J Immunol Res. 2021 Dec 30;2021:8356645. doi: 10.1155/2021/8356645 (PMC8739918; doi:10.1155/2021/8356645)

**Supplementary Materials**

**Lipopolysaccharide-induced transcriptional changes in LBP-deficient rat and its possible implications for the liver dysregulation during sepsis**

The PDF file includes:

Three supplementary tables: Table S1-3

Two supplementary figures: Figure S1-2

**Table S1. Primers for qPCR in the study.**

**Table S2. Number of uniquely mapped reads for each sample.**

**Table S3. The labelled DEGs of volcano plot between normal and LBP-deficient rats with the time of 0h, 6h, 24h respectively.**

**Figure S1. Boxplots of interested DEGs related to inflammatory response and lipid metabolic process between normal and LBP-deficient rats after LPS injection.**

**Figure S2. Boxplots of interested DEGs most associated with enriched GO terms and pathways.**

**Table S1. Primers for qPCR in the study.**

| Gene Name | Primer Sequence | Gene Name | Primer Sequence |
| --- | --- | --- | --- |
| GAPDH-F | AGTTCAACGGCACAGTCAAG | GAPDH-R | TACTCAGCACCAGCATCACC |
| Cxcl10-F | CCAAGTGCTGCTGTCGTTCTCTG | Cxcl10-R | GGTCTCAGCGTCTGTTCATGGAAG |
| Ifng-F | ACAACCCACAGATCCAGCACAAAG | Ifng-R | CACCGACTCCTTTTCCGCTTCC |
| Serpine1-F | GCGTCTTCCTCCACAGCCATTC | Serpine1-R | TGTCTCTGTTGGATTGTGCCGAAC |
| Eci1-F | CCGAGCGTGCCCTTCAACTG | Eci1-R | GCCATCACTGAGCGAGCCTTG |
| Dhrs7-F | CACCCAGACGACCATCCTACCC | Dhrs7b-R | CACATTCTTTGCCGAGACCTGAGG |
| Tysnd1-F | GCTGCTGGCTTGTGGCTCTC | Tysnd1-R | GAACACTCCGCCGCCTTCTG |

**Table S2. Number of uniquely mapped reads for each sample.**

| Samples | Total reads | Total mapped reads | Unique mapped reads | Multiple mapped reads | Unmapped reads |
| --- | --- | --- | --- | --- | --- |
| 0h_CTR1 | 56201412 | 52087146 (92.68%) | 43738274 (77.82%) | 3222006 (5.73%) | 4114266 (7.32%) |
| 0h_CTR2 | 64552167 | 60017967 (92.98%) | 50214439 (77.79%) | 3816957 (5.91%) | 4534200 (7.02%) |
| 0h_CTR3 | 61223804 | 57113467 (93.29%) | 46707009 (76.29%) | 3839244 (6.27%) | 4110337 (6.71%) |
| 0h_LBP1 | 87856886 | 81639073 (92.92%) | 66196887 (75.35%) | 5745978 (6.54%) | 6217813 (7.08%) |
| 0h_LBP2 | 60693699 | 56450271 (93.01%) | 46343556 (76.36%) | 3769966 (6.21%) | 4243428 (6.99%) |
| 0h_LBP3 | 59876807 | 55708883 (93.04%) | 45149264 (75.4%) | 3961284 (6.62%) | 4167924 (6.96%) |
| 6h_CTR1 | 57700971 | 53572812 (92.85%) | 44750929 (77.56%) | 3322452 (5.76%) | 4128159 (7.15%) |
| 6h_CTR2 | 68148171 | 63544958 (93.25%) | 53016978 (77.8%) | 4064767 (5.96%) | 4603213 (6.75%) |
| 6h_CTR3 | 58209518 | 52992355 (91.04%) | 43745868 (75.15%) | 3447599 (5.92%) | 5217163 (8.96%) |
| 6h_LBP1 | 71931200 | 66098399 (91.89%) | 53527154 (74.41%) | 4501085 (6.26%) | 5832801 (8.11%) |
| 6h_LBP2 | 66680569 | 61117600 (91.66%) | 49372024 (74.04%) | 4221627 (6.33%) | 5562969 (8.34%) |
| 6h_LBP3 | 73241006 | 67517908 (92.19%) | 55132382 (75.28%) | 4557696 (6.22%) | 5723098 (7.81%) |
| 24h_CTR1 | 64774170 | 59232196 (91.44%) | 47338976 (73.08%) | 4363264 (6.74%) | 5541974 (8.56%) |
| 24h_CTR2 | 102335602 | 95024478 (92.86%) | 73054344 (71.39%) | 7541018 (7.37%) | 7311124 (7.14%) |
| 24h_CTR3 | 62142838 | 57247482 (92.12%) | 45530167 (73.27%) | 4259389 (6.85%) | 4895356 (7.88%) |
| 24h_LBP1 | 107248623 | 99592430 (92.86%) | 80646853 (75.2%) | 6897368 (6.43%) | 7656193 (7.14%) |
| 24h_LBP2 | 60605986 | 55536266 (91.63%) | 45030293 (74.3%) | 3903645 (6.44%) | 5069720 (8.37%) |
| 24h_LBP3 | 70308603 | 64557988 (91.82%) | 51912712 (73.84%) | 4598265 (6.54%) | 5750615 (8.18%) |

**Table S3. The labelled DEGs of volcano plot between normal and LBP-deficient rats with the time of 0h, 6h, 24h respectively.**

| Category | | Genes | *P*-value | log2(FoldChange) | Regulation |
| --- | --- | --- | --- | --- | --- |
| 0 hour | WT | *Vdac2* | 1.5E-05 | -5.18 | DR |
|  |  | *RF00002* | 7.2E-05 | -6.66 | DR |
|  |  | *LOC259244* | 1.5E-04 | -5.07 | DR |
|  |  | *Pfn1* | 1.7E-04 | -6.30 | DR |
|  |  | *AC120071.1* | 1.7E-04 | -7.96 | DR |
|  |  | *Ube2c* | 2.8E-04 | -1.95 | DR |
|  |  | *Tars* | 3.5E-04 | -2.20 | DR |
|  | LBP^-/-^ | *Kat14* | 1.0E-05 | 1.62 | UR |
|  |  | *Gtf2i* | 1.3E-05 | 3.80 | UR |
|  |  | *Itih4* | 1.7E-05 | 9.19 | UR |
|  |  | *Rapgef4* | 3.4E-05 | 3.15 | UR |
|  |  | *Gorasp2* | 5.0E-05 | 3.82 | UR |
|  |  | *Rps6ka4* | 6.4E-05 | 2.09 | UR |
|  |  | *Matr3* | 6.8E-05 | 3.63 | UR |
|  |  | *Cyp2c7* | 6.8E-05 | 1.55 | UR |
|  |  | *Kif16b* | 7.5E-05 | 1.72 | UR |
|  |  | *Arhgap29* | 9.9E-05 | 2.88 | UR |
|  |  | *Lrp5* | 9.9E-05 | 3.32 | UR |
|  |  | *Tmem185b* | 1.3E-04 | 2.13 | UR |
|  |  | *Mrps10* | 1.3E-04 | 2.49 | UR |
|  |  | *Echdc1* | 1.7E-04 | 1.91 | UR |
|  |  | *Sds* | 1.9E-04 | 4.61 | UR |
|  |  | *Plekhf2* | 2.1E-04 | 2.48 | UR |
|  |  | *Set* | 2.2E-04 | 2.68 | UR |
|  |  | *Nufip2* | 2.6E-04 | 1.60 | UR |
|  |  | *Nr1d1* | 4.5E-04 | 4.17 | UR |
|  |  | *Cyp7a1* | 5.3E-04 | 3.32 | UR |
|  |  | *Usp2* | 9.0E-04 | 2.33 | UR |
| 6 hour | WT | *Ces2e* | 1.4E-06 | -3.92 | DR |
|  |  | *Edar* | 6.9E-06 | -1.71 | DR |
|  |  | *Csnk1a1* | 1.9E-05 | -1.53 | DR |
|  |  | *Pacsin3* | 3.7E-05 | -4.04 | DR |
|  |  | *RGD1562114* | 4.0E-05 | -2.09 | DR |
|  |  | *Cela1* | 5.2E-05 | -2.55 | DR |
|  |  | *AABR07063425.1* | 9.3E-05 | -8.45 | DR |
|  |  | *Ogfr* | 1.1E-04 | -3.25 | DR |
|  |  | *RGD1559960* | 1.4E-04 | -2.00 | DR |
|  |  | *Pabpc4* | 2.1E-04 | -3.03 | DR |
|  |  | *Trim36* | 2.2E-04 | -1.93 | DR |
|  |  | *Baiap2l1* | 2.7E-04 | -1.60 | DR |
|  |  | *Kdm4a* | 2.9E-04 | -2.74 | DR |
|  |  | *Nfkbiz* | 5.2E-04 | -1.73 | DR |
|  |  | *Rmdn3* | 6.8E-04 | -2.01 | DR |
|  |  | *Agxt2* | 7.9E-04 | -1.92 | DR |
|  |  | *Pdzk1ip1* | 8.0E-04 | -2.58 | DR |
|  | LBP^-/-^ | *Pacsin3* | 3.5E-07 | 4.62 | UR |
|  |  | *Eef1a1* | 3.8E-05 | 9.71 | UR |
|  |  | *LOC103694879* | 4.5E-05 | 2.99 | UR |
|  |  | *Pycr2* | 3.0E-04 | 1.54 | UR |
|  |  | *Sigmar1* | 3.2E-04 | 1.55 | UR |
|  |  | *Aip* | 4.3E-04 | 2.88 | UR |
|  |  | *LOC691083* | 4.5E-04 | 3.31 | UR |
|  |  | *Syne4* | 5.6E-04 | 1.71 | UR |
|  |  | *Cox20* | 6.2E-04 | 2.02 | UR |
|  |  | *Lyz2* | 6.2E-04 | 2.11 | UR |
|  |  | *Mob2* | 7.2E-04 | 2.65 | UR |
|  |  | *Ldb1* | 8.2E-04 | 1.52 | UR |
| 24 hour | WT | *Hao1* | 1.4E-05 | -5.81 | DR |
|  |  | *Slain2* | 4.4E-05 | -1.80 | DR |
|  |  | *Pik3ca* | 8.5E-05 | -2.05 | DR |
|  |  | *Csf1r* | 2.0E-04 | -3.91 | DR |
|  |  | *Tmem132a* | 2.6E-04 | -2.00 | DR |
|  |  | *Pomk* | 2.7E-04 | -2.23 | DR |
|  |  | *Trak2* | 3.7E-04 | -1.68 | DR |
|  |  | *RGD1310495* | 3.7E-04 | -2.23 | DR |
|  |  | *Fabp7* | 5.8E-04 | -3.80 | DR |
|  |  | *Cep8c9* | 6.3E-04 | -2.23 | DR |
|  |  | *Tmem183a* | 7.2E-04 | -3.60 | DR |
|  |  | *Acvr1* | 8.4E-04 | -2.22 | DR |
|  |  | *Itih4* | 9.5E-04 | -9.08 | DR |
|  | LBP^-/-^ | *AABR07031184.1* | 1.4E-05 | 5.27 | UR |
|  |  | *Glyatl1* | 7.8E-05 | 5.46 | UR |
|  |  | *Tmem176b* | 1.9E-04 | 4.27 | UR |
|  |  | *Timm9* | 2.2E-04 | 2.52 | UR |
|  |  | *Nudt6* | 6.2E-04 | 4.54 | UR |

**Figure S1. Boxplots of interested DEGs related to inflammatory response and lipid metabolic process between normal and LBP-deficient rats after LPS injection.**


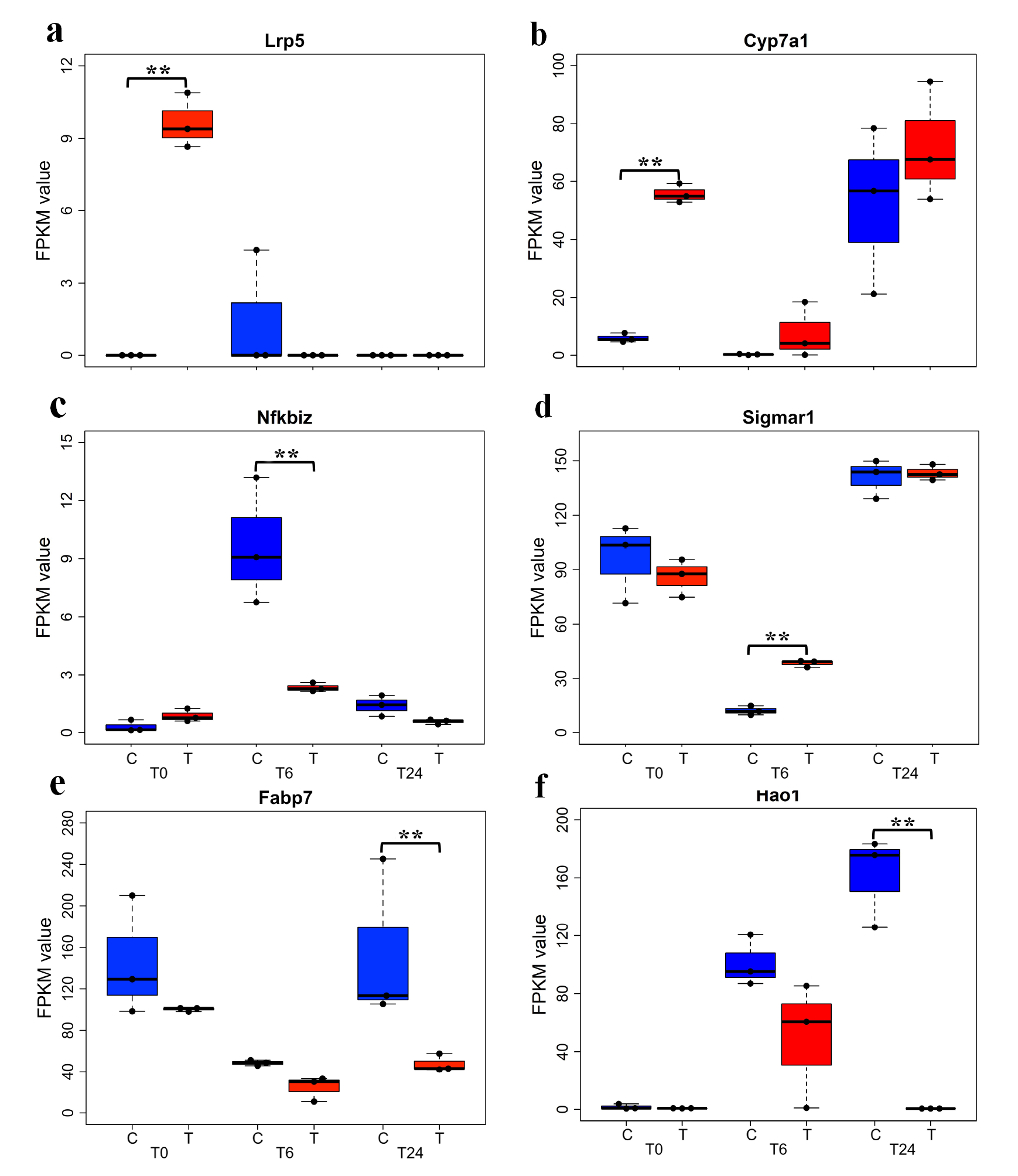


**Figure S2. Boxplots of interested DEGs most associated with enriched GO terms and pathways.**


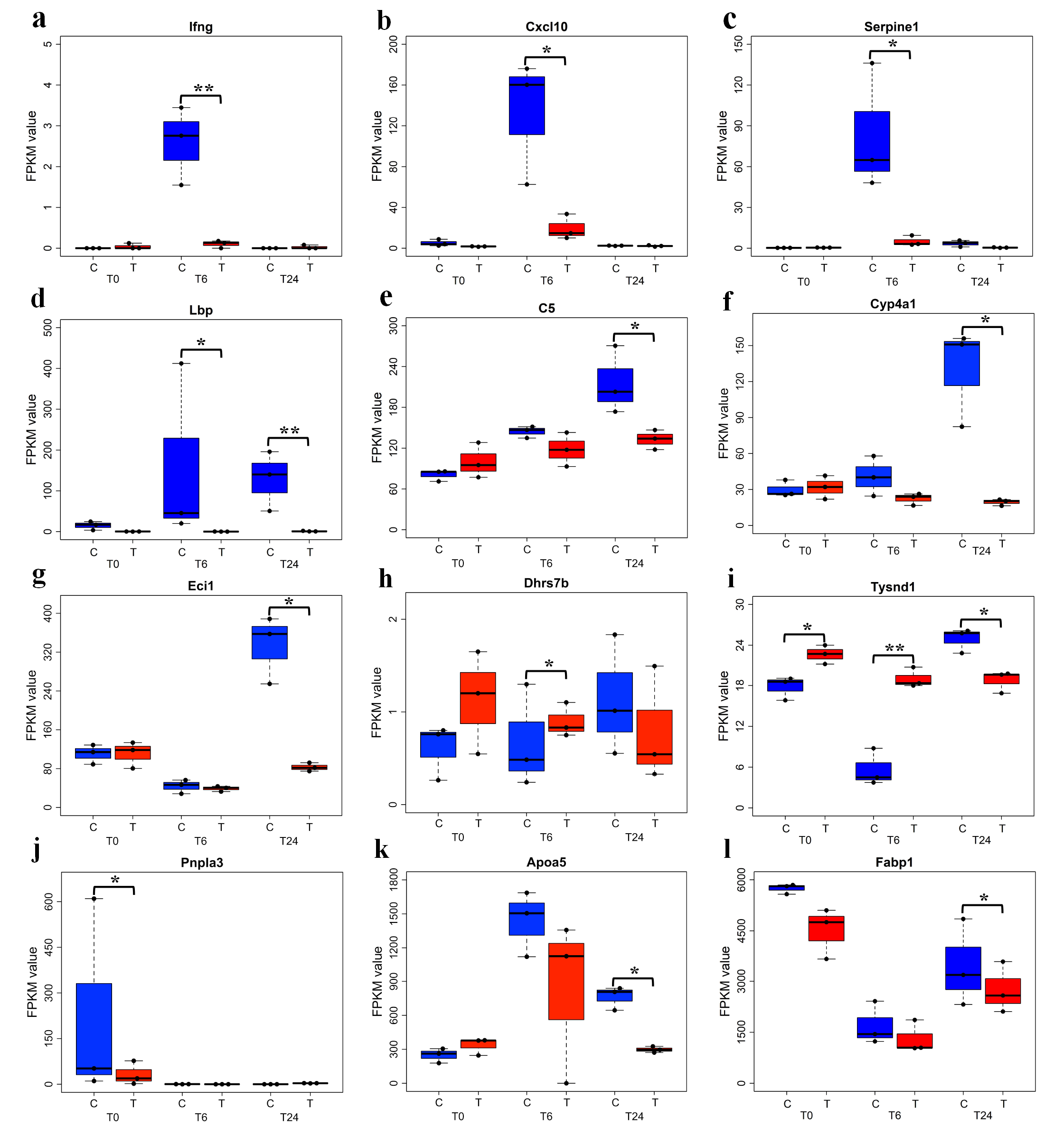

Supplement: Supplementary Materials — Supplementary Table S1: primers for qPCR in the study. Supplementary Table S2: number of uniquely mapped reads for each sample. Supplementary Table S3: the labeled DEGs of volcano plot between normal and LBP-deficient rats with the time of 0 h, 6 h, and 24 h, respectively. Supplementary Figure S1: boxplots of interested DEGs related to inflammatory response and lipid metabolic process between normal and LBP-deficient rats after LPS injection. Supplementary Figure S2: boxplots of interested DEGs most associated with enriched GO terms and pathways. [file 8356645.f1.docx]
